# Associations between stressors and leave-taking behavior among nursing interns: a cross-sectional quantitative survey

**DOI:** 10.3389/fmed.2026.1748069

**Published:** 2026-05-01

**Authors:** Huawen Song, Yanping Liu

**Affiliations:** Department of Nursing, Linfen People’s Hospital, Linfen, Shanxi, China

**Keywords:** academic stress, clinical internship, clinical stress, leave-taking behavior, nursing interns

## Abstract

**Objective:**

To examine the relationship between academic and clinical stressors and leave-taking frequency among nursing interns, and to identify independent predictors of frequent leave behavior.

**Methods:**

A cross-sectional study was conducted between June 2021 and April 2024 at Linfen People’s Hospital. Using a consecutive sampling method, 910 nursing interns were included. Baseline characteristics, leave records, and academic and stress scale data were collected. Group comparisons, correlation analyses, and ordinal logistic regression were performed.

**Results:**

Significant group differences were found in academic and clinical stress levels (*p* < 0.05). The high-frequency leave group reported greater stress from examinations, fear of failure, internship workload, and clinical skill application. Correlation analysis revealed a strong cross-domain link between Fear of Failure and Clinical Skills Application (*r* = 0.53, *p* < 0.001). Regression analysis identified several independent predictors, with clinical skill application and examination pressure showing the strongest effects.

**Conclusion:**

The findings demonstrate that academic and clinical stressors jointly contribute to leave-taking behavior among nursing interns, with a notable cross-domain spillover effect. Targeted interventions should be tailored for vulnerable subgroups.

## 1 Introduction

Clinical internship is a critical stage in nursing education, serving as an essential bridge between theoretical learning and practical application (1). In the Chinese nursing education system, students typically undertake a mandatory, comprehensive clinical internship lasting approximately 40 weeks after completing their theoretical coursework. During this intensive phase, students complete sequential rotations through core clinical departments. Under the dual supervision of hospital-appointed clinical preceptors and academic advisors from their educational institutions, interns are expected to apply theoretical knowledge, master fundamental nursing skills, navigate complex clinical environments, and begin developing a nascent professional identity (2). A high-quality internship not only enhances students’ clinical competence but also shapes their future professional trajectory (3). However frequent leave-taking (defined in this study as formally approved short-term time off) or clinical absenteeism (defined in this study as non-attendance) among nursing interns has become an emerging concern in recent years (4). Such interruptions in clinical placement may undermine the continuity of experiential learning and increase the workload and supervisory burden for clinical instructors (4, 5).

Absenteeism among nursing interns is rarely a matter of individual choice; rather, it results from the complex interplay of multiple influencing factors (6). Studies have shown that during the internship phase, nursing students are exposed to various sources of stress originating from both academic and clinical environments (7). Academic stress—including examination pressure, heavy coursework, and fear of academic failure—represent major sources of tension in nursing education (8). This sustained pressure not only impairs concentration and academic performance but can also trigger chronic anxiety, sleep disturbances, and a loss of motivation for learning (9). Clinical stressors are highly prevalent among nursing students. These include excessive workload, insufficient clinical skills, and challenging interactions with instructors and patients. Such stressors have been closely associated with anxiety, depression, burnout, and reduced self-efficacy (10–12). Excessive stress can negatively affect interns’ psychological well-being, learning performance, and professional identity, ultimately manifesting in behavioral outcomes such as absenteeism or even withdrawal from clinical practice (13, 14). In addition, certain contextual factors may further influence how stress is perceived and expressed through behavioral responses (15).

To date, while numerous studies have focused on the relationship between stress and mental health outcomes such as anxiety and depression, relatively few have systematically examined the association between stressors and leave-taking frequency among nursing interns. To address these gaps, the present study had three primary objectives: (1) to examine the relationship between academic and clinical stressors and leave-taking frequency among nursing interns; (2) to explore the interrelationships among these stressors; and (3) to identify the independent predictors of high-frequency leave-taking behavior. By integrating both academic and clinical dimensions, this study aims to provide a more comprehensive understanding of the mechanisms linking stress to behavioral outcomes during clinical training, offering evidence to inform targeted support strategies.

## 2 Methods

### 2.1 Study population

This study included a total of 910 nursing interns who completed their internships at Linfen People’s Hospital during the consecutive three-year period from June 2021 to April 2024. These interns hail from multiple nursing schools including Shanxi Management Vocational College, Shanxi Medical University, and Linfen Vocational and Technical College, with their birthplaces span provinces and municipalities across the country. The hospital admits and schedules nursing interns on an annual basis for approximately 40-week clinical rotations, with a six-month interval between cohorts. Among them, 389 interns (42.7%) had recorded leave during their internship, and their data were included in the subsequent analysis of leave-taking behavior. The inclusion criteria were as follows: (1) completed the required 40-week comprehensive clinical internship and rotated through all designated departments in accordance with the teaching plan; (2) aged ≤ 30 years; (3) had a good health status confirmed by physical examinations upon enrollment and before the internship, with no diseases or physical disabilities that would affect normal internship participation. The exclusion criteria were: (1) those who interrupted or failed to complete the entire internship program due to personal reasons; (2) those who were lost to follow-up before completing the required clinical rotation.

Based on the total number of leave days accumulated during the internship, all 910 interns were divided into three groups for subsequent analysis: Low-frequency group (0–3 days, *n* = 521), Medium-frequency group (4–7 days, *n* = 237), and High-frequency group (≥8 days, *n* = 152). This classification was guided by the hospital’s internship management regulations and was consistent with grouping approaches used in prior studies on people attendance patterns (16).

### 2.2 Data collection

Data in this study were collected through multiple channels. First, by reviewing the hospital’s onboarding records and combining with a self-designed Participant Demographic Questionnaire, information including participants’ name, gender, educational background, marital status, commute time, and family financial support was collected. Second, complete leave records of interns from three consecutive cohorts were obtained from the hospital’s Nursing Department, which included the number of leave days, reasons for leave, start and end dates, and relevant supporting documents. Among these, reasons for leave were uniformly categorized into five types during data entry: examination-related factors, physical condition, family reasons, job interview, and others.

### 2.3 Questionnaire survey

During the final week of clinical training, the research team developed a self-report stress questionnaire comprising two dimensions—the Academic Stressors Scale and the Clinical Stressors Scale—based on relevant literature, expert input, and the specific structure of nursing education and clinical training in China. Notably, the questionnaire was independently reviewed by three senior nursing experts, each with over 10 years of experience in nursing education and clinical supervision. Incorporating their written feedback, the research team revised the questionnaire and finalized its structure and items.

The Academic Stressors Scale was partially adapted from the Student Nurse Stress Index developed by Jones and Johnston (17), and partially informed by the work of Ding et al. (15). It consists of five items designed to assess stress related to the theoretical training phase. A 5-point Likert scoring system was applied. Each item was rated according to the perceived level of stress: “Not stressed” = 1 point, “Moderately stressed” = 2 points, “Neutral” = 3 points, “Stressed” = 4 points, and “Highly stressed” = 5 points. The total score was calculated by summing the scores of all items. The total score system is: scores ranging from 5 to 11 were categorized as low stress, 12 to 28 as moderate stress, and 19 to 25 as high stress. The Clinical Stressors Scale, adapted from Mohamed et al. (7) to assess stress experienced by students in the clinical practice environment, comprises six items and employs an identical scoring system, with scores of 6 to 13 indicating low stress, 14 to 26 indicating moderate stress, and 22 to 30 indicating high stress.

Prior to use, the content validity of both scales was validated using the Delphi method. Expert inclusion criteria were: (1) having over 10 years of work experience in the nursing field; (2) possessing recognized expertise in their field, such as publishing academic papers, holding leadership positions, or having professional reputation; (3) voluntarily participating in this study. A total of 20 experts were invited to participate in two rounds of expert consultation. The expert authority coefficient was 0.89, with a 100% response rate for both rounds. The consensus standard was defined as a coefficient of variation (CV) < 0.25. The degree of alignment in expert opinions across the two rounds is shown in Table 1. Two rounds of expert consultation revealed that all scales met consensus standards, demonstrating good content validity. In the present study, both questionnaires demonstrated good internal consistency reliability, with Cronbach’s *α* coefficients of 0.82 for the Academic Stressors Scale and 0.79 for the Clinical Stressors Scale. The detailed scales are shown in Table 2.

**Table 1:** The degree of coordination of expert opinions.

Item	Importance		Operability	
	CV	Kendall’s *W*	CV	Kendall’s *W*
Academic stressors scale				
First round	0.152	0.178	0.146	0.169
Second round	0.071	0.318	0.064	0.291
Clinical stressors scale				
First round	0.148	0.182	0.142	0.172
Second round	0.069	0.329	0.061	0.302

**Table 2:** The academic stressors scale and the clinical stressors scale.

Stressor	Not stressed	Moderately stressed	Neutral	Stressed	Highly stressed
Academic stressors scale					
1. Stress associated with the volume and pace of assigned coursework and readings (Course Load)					
2. Stress arising from the complexity and intellectual challenge of the curriculum (Course Difficulty)	1	2	3	4	5
3. Pressure related to formal assessments and the need to achieve satisfactory academic performance (Examinations and Grades)	1	2	3	4	5
4. Anxiety about not meeting academic expectations or the possibility of failing courses (Fear of Failure)	1	2	3	4	5
5. Stress or uncertainty regarding institutional standards, graduation prerequisites, and academic demands (Academic Requirements)	1	2	3	4	5
Clinical stressors scale					
1. Stress related to communication, feedback, and the overall dynamic with clinical supervisors or mentors (Interaction with Teachers)	1	2	3	4	5
2. Stress associated with interactions and establishing rapport with practicing nurses and other healthcare team members (Relationship with Clinical Staff)	1	2	3	4	5
3. Stress perceived from the overall learning environment, supportiveness, and culture within the clinical setting (Clinical Teaching Atmosphere)	1	2	3	4	5
4. Stress resulting from the amount, intensity, and pace of clinical duties and responsibilities (Internship Workload)	1	2	3	4	5
5. Anxiety or pressure experienced when applying learned theoretical knowledge and skills to actual patient care situations (Clinical Skill Application)	1	2	3	4	5
6. Stress stemming from communication, managing relationships, and addressing the needs of patients (Interaction with Patients)	1	2	3	4	5

### 2.4 Statistical analysis

Statistical analyses were performed using SPSS version 25.0. Continuous variables are described as mean ± standard deviation, while categorical variables are presented as frequencies and percentages (n, %). Group comparisons were conducted using one-way ANOVA (with post-hoc comparisons employing the Bonferroni correction) or the Chi-square test, as appropriate. Pearson correlation coefficients were computed to examine bivariate relationships among demographic characteristics, academic stressors, and clinical stressors. For ordinal logistic regression analysis, the proportional odds assumption was formally tested using the Brant test. A multivariate ordinal logistic regression model was subsequently employed to identify independent predictors of leave-taking frequency, adjusting for gender, age, marital status, and educational background. Results are presented as odds ratios (OR) with their corresponding 95% confidence intervals (CI). The significance level was set at *p* < 0.05 for all analyses.

### 2.5 Ethical approval

This study was approved by the Ethics Committee of Linfen People’s Hospital(Approval No: LFPH-NTR-202113). Students were invited to participate voluntarily in the research. All participants were informed that their involvement was entirely voluntary, and each individual retained the right to accept or decline participation.

## 3 Results

### 3.1 Leave condition and demographic characteristics of nursing interns

**Figure 1:**
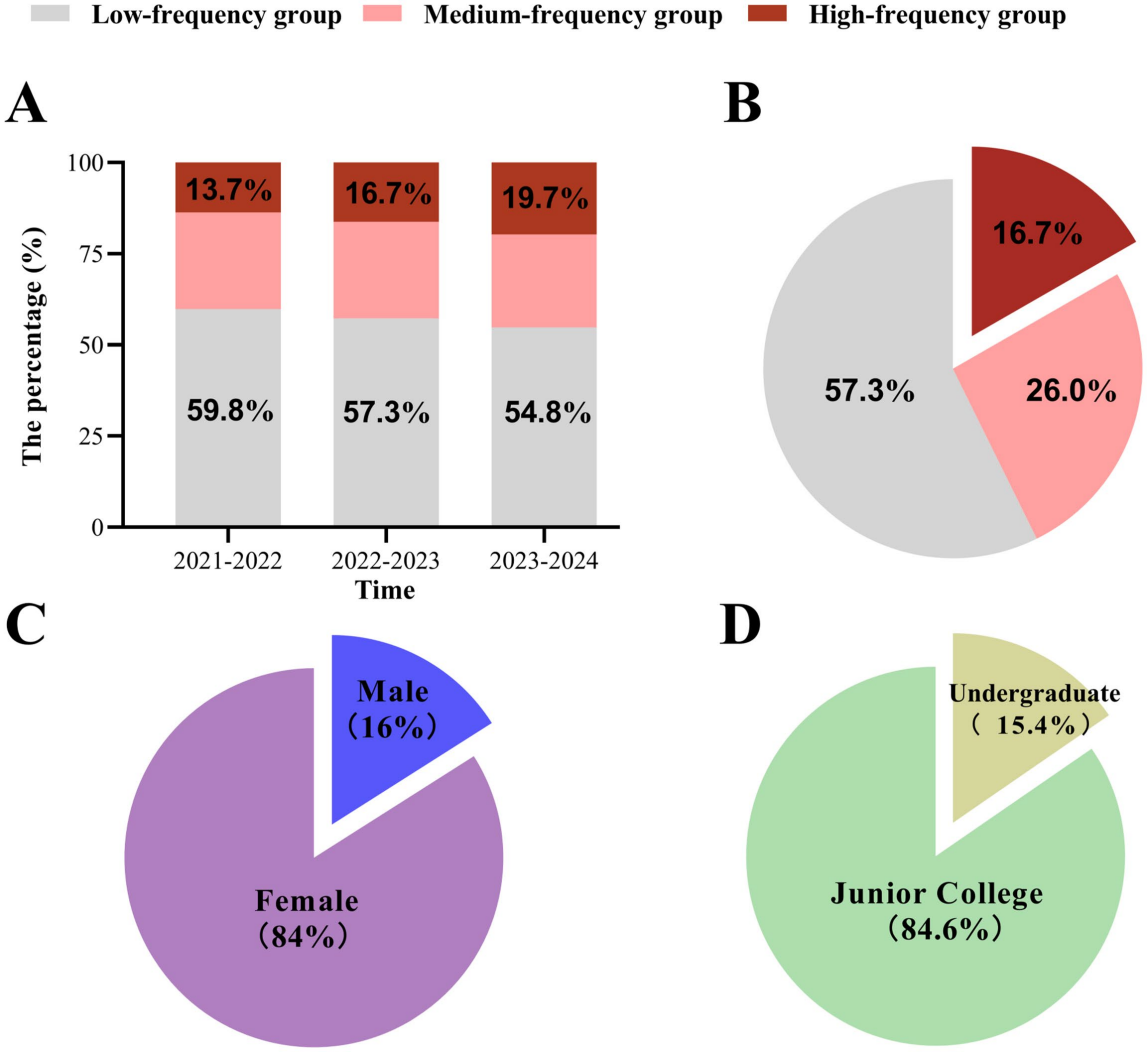
Leave longitudinal trends, frequency distribution, and demographic profile. **(A)** Longitudinal trend in the proportion of each leave frequency group across three intern cohorts. **(B)** Overall distribution of nursing interns by leave frequency group (*N* = 910). **(C)** Gender distribution of the sample. **(D)** Distribution of the sample by educational background.

Trend analysis across three consecutive intern cohorts revealed a key finding: the proportion of interns in the high-frequency leave group demonstrated a clear and year-on-year increase, rising from 13.7% in the 2021–2022 cohort to 16.7% in the 2022–2023 cohort, and reaching 19.7% in the 2023–2024 cohort (Figure 1A). Among all 910 interns, over half (57.3%) belonged to the low-frequency leave group, while the medium-frequency and high-frequency groups accounted for 26.0 and 16.7%, respectively (Figure 1B). Demographically, the sample was predominantly composed of female interns (84%) and those with a junior college educational background (84.6%) (Figures 1C,D).

The distribution of leave reasons among the three intern groups is presented in Table 3. Overall, examination-related factors were the primary reason for leave (62.75%), followed by physical condition (21.87%) and family reasons (11.54%). The Chi-square test indicated that the distribution of leave reasons did not differ significantly among the three groups (*χ*^2^ = 0.224, *p* = 0.894).

**Table 3:** Distribution of leave reasons among nursing interns.

Reasons	Total	Low-frequency group (*n* = 521)	Medium-frequency group (*n* = 237)	High-frequency group (*n* = 152)
Examination-related	571 (62.75%)	328 (62.95%)	149 (62.87%)	94 (61.84%)
Physical condition	199 (21.87%)	111 (21.30%)	54 (22.78%)	34 (22.37%)
Family reasons	105 (11.54%)	59 (11.33%)	28 (11.81%)	18 (11.84%)
Job interview	27 (2.97%)	15 (2.88%)	7 (2.95%)	5 (3.29%)
Others	8 (0.87%)	5 (0.96%)	2 (0.84%)	1 (0.66%)

Data are presented as *n* (%). Differences in the distribution of reasons across groups were assessed by the Chi-square test and were not statistically significant (*p* > 0.05).

### 3.2 Association between demographic factors and leave frequency groups

**Figure 2:**
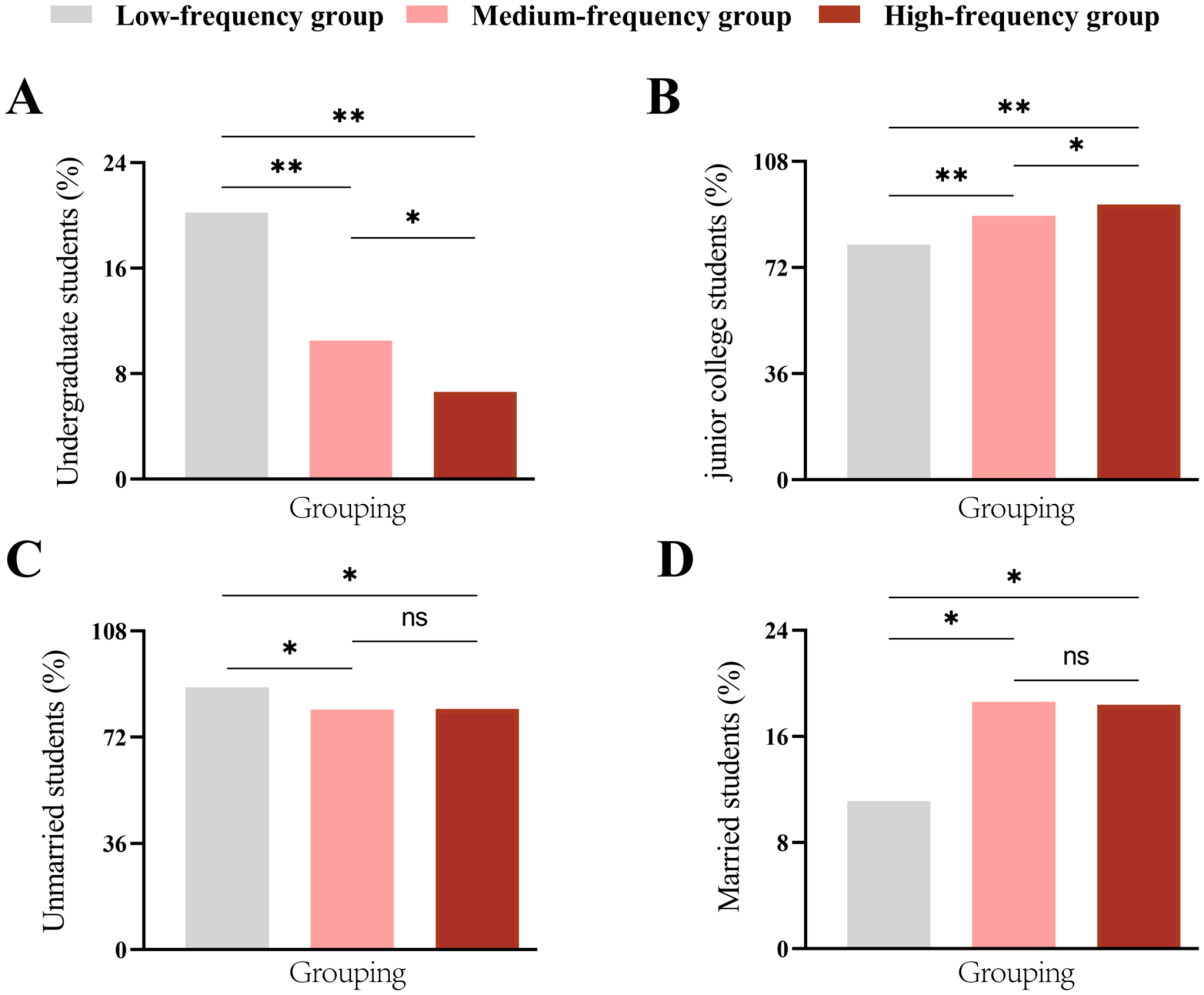
*Post hoc* comparisons of educational background and marital status across leave frequency groups. **(A,B)** Proportion of interns with undergraduate or junior college background across leave frequency groups. **(C,D)** Proportion of unmarried or married interns across leave frequency groups. Significance codes: *p < 0.05, **p < 0.01; ns, not significant.

Univariate analysis revealed that the distributions of educational background and marital status differed significantly across the leave frequency groups (*p* < 0.05), while no statistically significant differences were found for age, gender, commute time, school, birthplaces, or family financial support (*p* > 0.05) (Table 4). Post-hoc pairwise comparisons were further conducted for educational background and marital status. The results for educational background demonstrated that the proportion of interns from a junior college background showed significant pairwise differences between the low-, medium-, and high-frequency leave groups (*p* < 0.05), revealing a trend where the percentage of junior college students increased significantly with each ascending level of leave frequency (Figures 2A,B). Concurrently, the prevalence of married status was significantly higher in both the medium- (18.6%) and high-frequency (18.4%) groups compared to the low-frequency group (11.1%) (*p* < 0.05), with no significant difference between the two higher-frequency groups (Figures 2C,D).

**Table 4:** Comparison of baseline characteristics across leave frequency groups.

Variable	Low-frequency group (*n* = 521)	Medium-frequency group (*n* = 237)	High-frequency group (*n* = 152)	*χ*^2^/*F*	*P*
Age, (mean ± SD)	19.9 ± 1.2	20.0 ± 1.3	19.8 ± 1.1	1.248	0.289
Gender, n (%)				1.371	0.502
Male	90 (17.31)	34 (14.28)	22 (14.54)		
Female	431 (82.69)	203 (85.72)	130 (85.46)		
Educational Background, *n* (%)				22.414	<0.001
Undergraduate	105 (20.20)	25 (10.47)	10 (6.68)		
Junior College	416 (79.80)	212 (89.53)	142 (93.32)		
Birthplaces				4.892	0.798
Shanxi Province	230 (44.15)	104 (43.88)	66 (43.42)		
Henan Province	115 (22.07)	52 (21.94)	33 (21.71)		
Hebei Province	93 (17.85)	42 (17.72)	28 (18.42)		
Inner Mongolia Autonomous Region	53 (10.17)	25 (10.55)	16 (10.53)		
Others	30 (5.76)	14 (5.91)	9 (5.92)		
Schools				9.847	0.455
Shanxi University of Chinese Medicine	50 (9.60)	20 (8.44)	8 (5.26)		
Linfen Vocational And Technical College	178 (34.16)	82 (34.60)	52 (34.21)		
Shanxi Health Vocational College	100 (19.19)	45 (18.99)	28 (18.42)		
Yuncheng Vocational Nursing College	93 (17.85)	42 (17.72)	28 (18.42)		
Others	100 (19.20)	48 (20.25)	36 (23.69)		
Marital Status, *n* (%)				9.867	0.007
Unmarried	463 (88.93)	193 (81.46)	124 (81.64)		
Married	58 (11.07)	44 (18.54)	28 (18.36)		
Commute time, *n* (%)				3.524	0.181
≤40 min	368 (70.59)	154 (65.0)	98 (64.47)		
>40 min	153 (29.41)	83 (35.0)	54 (35.53)		
Family financial support, *n* (%)				0.052	0.955
No	345 (66.17)	155 (65.4)	100 (65.81)		
Yes	176 (33.83)	82 (34.6)	52 (34.19)		

Values are presented as mean ± standard deviation or *n* (%). *p*-values for continuous variables were derived from one-way ANOVA, and for categorical variables from the Chi-square test. A *p*-value < 0.05 was considered statistically significant.

### 3.3 Academic and clinical stressors across leave frequency groups

Significant differences in academic stressor items were observed among the three leave frequency groups (Table 5). ANOVA results indicated statistically significant differences for Examinations and Grades (*F* = 296.76, *p* < 0.001) and Fear of Failure (*F* = 125.48, *p* < 0.001), with the high-frequency leave group scoring the highest, suggesting that academic evaluation and fear of failure are primary stressors associated with increased leave frequency. A significant difference was also found for Uncertainty about Academic Demands. In contrast, no statistically significant differences were detected for Amount of Classwork or Difficulty of Classwork.

**Table 5:** Comparison of academic stressor scores across leave frequency groups.

Academic load items	Low-frequency group	Medium-frequency group	High-frequency group	*F*	*P*
Course Load	3.12 ± 0.88	3.21 ± 0.52	3.18 ± 0.13	1.92	0.147
Course Difficulty	3.41 ± 0.67	3.36 ± 0.81	3.50 ± 0.27	2.067	0.127
Examinations and Grades	3.01 ± 0.91	3.45 ± 0.86	4.91 ± 0.54	296.7	<0.001
Fear of Failure	3.56 ± 0.81	3.90 ± 0.76	4.66 ± 0.53	125.5	<0.001
Academic Requirements	3.14 ± 0.79	3.20 ± 0.54	3.29 ± 0.24	3.46	0.033

Scores are presented as mean ± standard deviation, reflecting perceived stress levels on a 5-point Likert scale. Between-group differences were assessed by one-way ANOVA, with the corresponding *F* and *p*-values shown.

Moreover, analysis of clinical stressor items revealed significant variations across the groups (Table 6). The most pronounced differences were identified for Internship Workload (*F* = 37.52, *p* < 0.001) and Clinical Skill Application (*F* = 53.18, *p* < 0.001), where the high-frequency leave group again reported the highest scores, indicating that clinical work overload and skill-related pressure are significant stressors linked to leave behavior. Additionally, significant differences were found for Interaction with Instructors and Interaction with Patients.

**Table 6:** Comparison of clinical stressor scores across leave frequency groups.

Clinical stress items	Low-frequency group	Medium-frequency group	High-frequency group	*F*	*P*
Interaction with Teachers	2.89 ± 0.85	3.25 ± 0.78	3.40 ± 0.54	26.52	<0.001
Relationship with Clinical Staff	3.08 ± 0.96	3.10 ± 0.87	3.11 ± 0.72	0.08	0.923
Clinical Teaching Atmosphere	3.15 ± 0.88	3.18 ± 0.63	3.20 ± 0.49	0.23	0.795
Internship Workload	3.55 ± 0.92	3.65 ± 0.84	4.25 ± 0.59	37.52	<0.001
Clinical Skill Application	3.34 ± 0.87	3.70 ± 0.73	4.15 ± 0.65	53.18	<0.001
Interaction with Patients	3.20 ± 0.66	3.42 ± 0.89	3.75 ± 0.72	21.89	<0.001

Scores are presented as mean ± standard deviation, reflecting perceived stress levels on a 5-point Likert scale. Between-group differences were assessed by one-way ANOVA, with the corresponding *F* and *p*-values shown.

### 3.4 Correlations among key demographic and stressor variables

**Figure 3:**
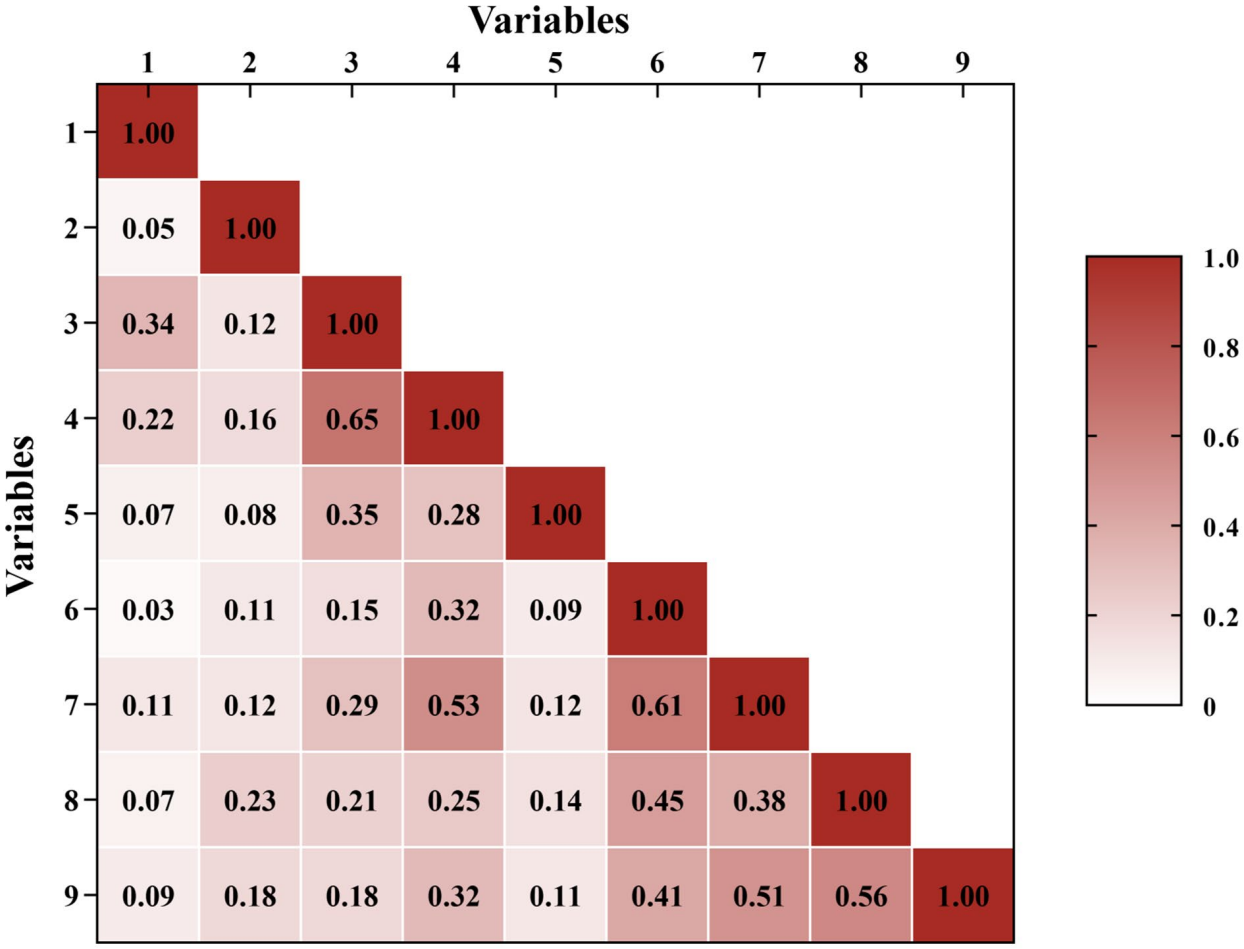
Correlation heatmap between different stress variables. In the heatmap, variables are numbered as follows: (1) Educational background, (2) Marital status, (3) Examinations and grades, (4) Fear of failure, (5) Academic requirements, (6) Internship workload, (7) Clinical skills application, (8) Interaction with teachers, (9) Interaction with patients. Stronger positive correlations are represented by darker shades of red, while negative or weaker associations appear lighter.

To clarify the internal relationships among stress-related variables, a correlation matrix including demographic characteristics, academic stress, and clinical stress factors was constructed (Table 7). The correlation structure was visualized through a heatmap (Figure 3).

**Table 7:** Correlations among key demographic and stressor variables.

Variable	1	2	3	4	5	6	7	8	9
1. Education	1								
2. Marital Status	0.050	1							
3. Examinations and Grades	0.340**	0.122*	1						
4. Fear of Failure	0.223**	0.163**	0.654***	1					
5. Academic Requirements	0.070	0.080	0.354**	0.284**	1				
6. Internship Workload	0.030	0.105*	0.153**	0.324**	0.090	1			
7. Clinical Skills Application	0.113*	0.117*	0.288**	0.533***	0.120	0.613***	1		
8. Interaction with Teachers	0.066	0.228**	0.210**	0.251**	0.140	0.454***	0.382**	1	
9. Interaction with Patients	0.091	0.179**	0.181*	0.322***	0.110	0.407***	0.509***	0.557 ***	1

*p < 0.05, **p < 0.01, ***p < 0.001; ns, not significant.

Regarding demographic factors, Education was positively correlated with Examinations and Grades as well as Fear of Failure (*r* = 0.34, 0.22, *p* < 0.01), indicating that interns with associate degrees perceived greater examination pressure. Marital Status showed multiple significant positive correlations, particularly with Interaction with Teachers (*r* = 0.23, *p* < 0.01), Fear of Failure (*r* = 0.16, *p* < 0.01), and Interaction with Patients (*r* = 0.18, *p* < 0.01). Within academic stressors, Fear of Failure was strongly associated with Examinations and Grades (*r* = 0.65, *p* < 0.001) and moderately with Academic Requirements (*r* = 0.28, *p* < 0.01), forming a cohesive academic stress cluster. Within clinical stressors, Internship Workload, Clinical Skills Application, and Interaction with Patients were moderately to strongly intercorrelated (*r* = 0.41–0.61, *p* < 0.001), suggesting a unified clinical workload domain. Importantly, a cross-domain stress spillover was observed: Fear of Failure exhibited a strong correlation with Clinical Skills Application (*r* = 0.53, *p* < 0.001).

### 3.5 Ordinal logistic regression analysis of factors influencing leave frequency

Ordinal logistic regression was conducted to identify independent predictors of leave-taking frequency among nursing interns (Table 8). Educational Background (OR = 2.06, *p* = 0.001) and Marital Status (OR = 1.71, *p* = 0.004) were both associated with higher leave frequency, indicating that interns with associate degrees and married status were more likely to take frequent leave. Regarding stress-related factors, Examinations and Grades (OR = 2.00, *p* < 0.001), Fear of Failure (OR = 1.84, *p* = 0.001), Internship Workload (OR = 1.75, *p* = 0.001), Clinical Skills Application (OR = 2.32, *p* < 0.001), and Interaction with Teachers (OR = 1.36, *p* = 0.047) were significant independent predictors. Among them, Clinical Skills Application and Examinations and Grades exerted the strongest effects on leave frequency. In contrast, Academic Requirements and Interaction with Patients were not significant predictors (*p* > 0.05). These results suggest that both academic and clinical stress domains contribute to leave-taking behavior.

**Table 8:** Ordinal logistic regression analysis of factors associated with leave frequency.

Variable	*β*	SE	Wald *χ*^2^	OR (95% CI)	*p*
Educational Background	0.724	0.216	11.20	2.06 (1.34–3.16)	0.001
Marital Status	0.538	0.189	8.11	1.71 (1.18–2.48)	0.004
Examinations and Grades	0.693	0.152	20.76	2.00 (1.49–2.69)	<0.001
Fear of Failure	0.612	0.178	11.81	1.84 (1.30–2.62)	0.001
Academic Requirements	0.174	0.143	1.48	1.19 (0.90–1.58)	0.224
Internship Workload	0.563	0.167	11.32	1.75 (1.28–2.41)	0.001
Clinical Skills Application	0.842	0.176	22.73	2.32 (1.64–3.27)	<0.001
Interaction with Teachers	0.306	0.154	3.96	1.36 (1.00–1.85)	0.047
Interaction with Patients	0.287	0.162	3.15	1.33 (0.97–1.83)	0.076

Model statistics: *χ*^2^(9) = 128.4, *p* < 0.001; Nagelkerke *R*^2^ = 0.324; Proportional odds assumption satisfied (*p* = 0.361).

## 4 Discussion

This cross-sectional study systematically investigated the relationship between stressors and leave-taking behavior among 910 nursing interns. Our findings reveal that both academic and clinical stressors are significantly associated with increased leave frequency. Key independent predictors identified include Educational Background, Marital Status, Examinations and Grades, Fear of Failure, Internship Workload, Clinical Skills Application, and Interaction with Teachers. These results underscore the multifaceted nature of stress in influencing internship attendance and highlight specific domains where targeted support may be most effective.

Academic stress remains a prevalent and well-documented challenge in nursing education. This study further elucidates how its mechanisms vary across students with different educational backgrounds. We found that “examinations and grades” and “fear of failure” were not only key academic stressors associated with frequent leave-taking but also significant independent predictors in the model (OR = 2.00 and 1.84, respectively), a finding consistent with prior literature (7, 17). Notably, interns with junior college reported significantly higher stress levels related to “examinations and grades” and “fear of failure” than their undergraduate counterparts, and these two stressors were highly correlated. This phenomenon may stem from a dual structural burden. On one hand, associate-degree students may have a relatively weaker foundation in theoretical knowledge and coping strategies, making them more prone to frustration and avoidance when facing academic assessments (18). On the other hand, the prevailing requirement of a bachelor’s degree as the minimum entry criterion in the healthcare job market places junior college graduates at a systemic disadvantage, amplifying career uncertainty (8). This structural dilemma intensifies their fear of failure—transforming exams from mere academic evaluations into perceived gatekeepers to career entry. Consequently, the interplay of knowledge gaps and institutional barriers exacerbates the psychological burden on associate-degree interns, not only increasing leave-taking behavior but also profoundly impacting their professional identity and mental health (18, 19). Therefore, collaborative efforts between educational institutions and hospitals are essential to implement supportive interventions—such as personalized academic guidance and coping skills training—that empower students to explore diverse career pathways, make informed vocational choices, and strengthen psychological resilience and professional identity, while also promoting more inclusive recruitment and career development policies (20).

Regarding clinical stressors, this study identified internship workload, clinical skills application, and interaction with instructors as significant independent predictors of higher leave frequency, corroborating existing evidence on the relationship between clinical pressure and absenteeism among nursing interns (21, 22). Nursing work is typically characterized by high intensity (23, 24). Due to heavy workloads, time pressures, and staff shortages, nursing interns often need to work overtime frequently to complete tasks, which may lead to fatigue and physical exhaustion (25, 26), thereby necessitating leave to recover or attend to personal matters. Meanwhile, anxiety regarding clinical skill proficiency may evoke fear of making errors or receiving negative evaluations, thus triggering avoidance behaviors (6, 27). A study conducted in the Chinese context found that Chinese nursing students were more likely to adopt avoidance behaviors when facing stress during clinical practice (28). Furthermore, unsupportive or critical interactions with clinical instructors can exacerbate perceived stress, reduce interns’ confidence, and increase their tendency to withdraw from the training environment (29). Notably, these clinical stressors demonstrated moderate-to-strong intercorrelations, suggesting a mutually reinforcing dynamic that may create an amplified stress experience. Specifically, heavy workload intensifies strained supervisory relationships, which in turn exacerbates anxiety regarding clinical skill application—collectively increasing the likelihood of frequent leave-taking (11, 30). To mitigate this compounding effect, it is essential to implement structured interventions such as reasonable workload allocation, mentorship programs that foster supportive instructor–intern communication, and simulated training to enhance clinical confidence in a low-stakes environment (31).

Building on the identified academic and clinical stressors, this study further reveals a significant cross-domain “stress spillover” effect between these two dimensions. Specifically, the strong correlation between fear of failure and clinical skills application indicates that academic anxiety may translate into clinical self-doubt, thereby triggering avoidance behaviors such as leave-taking (32, 33). This phenomenon can be understood through self-efficacy theory, as diminished academic confidence directly impairs perceived clinical competence, creating a cross-situational negative cycle (34–36). To address this mechanism, it is essential to integrate academic counseling with clinical mentoring through comprehensive interventions that help students break the negative linkage between academic and clinical self-perception (31). Beyond stress mechanisms, sociodemographic factors such as marital status also significantly influence leave-taking behavior. Married interns reported higher stress perception and leave frequency than their unmarried peers, likely due to greater role conflict and family responsibilities. This finding aligns with studies by Rayan (37) and Ballester-Ferrando et al. (38), suggesting that managerial practices should address diverse student needs by offering more flexible scheduling and targeted support for married students. Furthermore, examination-related emerged as the most common reason for leave. This prevalent phenomenon reflects that some students have not yet established the awareness that clinical practice reinforces and consolidates theoretical knowledge, perceiving only a time conflict between internship and exam preparation (39). Therefore, educators should strengthen the integration of theory and practice in curriculum design—for instance, by embedding key examination content into clinical rotations—to guide students in recognizing the clinical environment as an applied setting for theoretical learning, thereby alleviating role conflict and optimizing the synergy between internship and academic pursuits (40, 41).

To address the dual academic and clinical pressures faced by nursing interns, educational institutions should adopt a systematic approach that enhances students’ comprehensive competencies and coping abilities through both theoretical instruction and practical training. In practical training, emphasis should be placed on strengthening systematic instruction in nursing procedures. Modern teaching tools such as smart simulation systems should be actively incorporated to enhance students’ practical skills through high-fidelity training (42). One-on-one personalized coaching should be provided to students with weaker procedural skills to bridge skill gaps and boost their clinical confidence. Theoretical instruction should appropriately increase the weight of courses on nursing ethics and humanistic care (43). Interactive teaching methods like case studies and role-playing should be integrated to guide students in deeply understanding the essence of the nursing profession and establishing sound professional values. Simultaneously, experiential learning should simulate real clinical scenarios, allowing students to familiarize themselves with the work environment and common situations before entering clinical practice, thereby reducing psychological stress caused by unfamiliarity (44). Prior to clinical placement, systematic pre-placement training should be organized. This training should cover hospital orientation, familiarization with regulations and policies, and intensive practice of nursing procedures. This helps nursing interns fully understand placement requirements and prepare psychologically (45). During clinical practice, instructors should prioritize root-cause-oriented guidance, emphasizing effective communication with students to promptly identify their psychological state and sources of stress. This enables timely support in alleviating pressure and resolving practical issues. Teaching methods may incorporate engaging and interactive elements tailored to student preferences, enhancing their identification with and commitment to clinical nursing work (46). This approach strengthens self-efficacy and reduces psychological burdens stemming from academic and clinical demands. Additionally, in daily teaching, instructors should monitor students’ emotional fluctuations, encourage positive emotional management strategies, and guide them in developing healthy coping mechanisms. This continuous cultivation of psychological resilience fundamentally reduces stress levels, facilitating smooth adaptation and successful completion of clinical internships.

This study is subject to several limitations. The reliance on self-report measures for stress assessment may introduce potential recall bias or social desirability effects. Furthermore, as participants were recruited from a single institution, the generalizability of our findings to other healthcare settings or regional contexts may be limited. Future studies should address these constraints through multi-center designs involving diverse clinical environments to enhance external validity. The incorporation of objective measures—such as biometric indicators of stress or independent assessments of clinical performance—would complement self-reported data and provide a more robust understanding of these relationships. Qualitative approaches are also recommended to gain deeper contextual insights into interns lived experiences and coping strategies.

## Data Availability

The original contributions presented in the study are included in the article/Supplementary material, further inquiries can be directed to the corresponding author.
