# Correction: Associations between stressors and leave-taking behavior among nursing interns: a cross-sectional quantitative survey

**DOI:** 10.3389/fmed.2026.1888116

**Published:** 2026-06-23

**Authors:** Huawen Song, Yanping Liu

**Affiliations:** Department of Nursing, Linfen People's Hospital, Linfen, Shanxi, China

**Keywords:** academic stress, clinical internship, clinical stress, leave-taking behavior, nursing interns

The **Ethics statement** was erroneously given as:

This study was approved by the Ethics Committee of Linfen People's Hospital (Approval No: LFPH-NTR-202113). Students were invited to participate voluntarily in the research. All participants were informed that their involvement was entirely voluntary, and each individual retained the right to accept or decline participation.

The correct **Ethics statement** is:

This study was approved by the Ethics Committee of Linfen People's Hospital (Approval No.: E20260410001). Students were invited to participate voluntarily in the research. All participants were informed that their involvement was entirely voluntary, and each individual retained the right to accept or decline participation.

The original version of this article has been updated.

