# Supplementary material for: Associations between stressors and leave-taking behavior among nursing interns: a cross-sectional quantitative survey
Source: Front Med (Lausanne). 2026 May 1;13:1748069. doi: 10.3389/fmed.2026.1748069 (PMC13175823; doi:10.3389/fmed.2026.1748069)
Supplement: Supplementary file 1 [file Table_1.DOCX]

| **Variable** | **1** | **2** | **3** | **4** | **5** | **6** | **7** | **8** | **9** |
| --- | --- | --- | --- | --- | --- | --- | --- | --- | --- |
| **1. Education** | 1 |  |  |  |  |  |  |  |  |
| **2. Marital Status** | 0.050 | 1 |  |  |  |  |  |  |  |
| **3. Examinations and Grades** | **0.340**  ** | **0.122**  * | 1 |  |  |  |  |  |  |
| **4. Fear of Failure** | 0.223  ** | **0.163**  ** | **0.654**  *** | 1 |  |  |  |  |  |
| **5. Academic Requirements** | 0.070 | 0.080 | **0.354**  ** | **0.284**  ** | 1 |  |  |  |  |
| **6. Internship Workload** | 0.030 | **0.105**  * | **0.153**  ** | **0.324**  ** | 0.090 | 1 |  |  |  |
| **7. Clinical Skills Application** | **0.113**  * | **0.117**  * | **0.288**  ** | **0.533**  *** | 0.120 | **0.613**  *** | 1 |  |  |
| **8.** **Interaction with Teachers** | 0.066 | **0.228**  ** | **0.210**  ** | **0.251**  ** | 0.140 | **0.454**  *** | **0.382**  ** | 1 |  |
| **9.** **Interaction with Patients** | 0.091 | **0.179**  ** | **0.181*** | **0.322**  *** | 0.110 | **0.407**  *** | **0.509**  *** | **0.557**  *** | 1 |

**Table1. Correlations Among Key Demographic and Stressor Variables**

Note: Significance codes: **p* < 0.05, ***p* < 0.01; ****p* < 0.001; ns, not significant.
